# Degradable Nanogels Based on Poly[Oligo(Ethylene Glycol) Methacrylate] (POEGMA) Derivatives through Thermo-Induced Aggregation of Polymer Chain and Subsequent Chemical Crosslinking

**DOI:** 10.3390/polym16081163

**Published:** 2024-04-20

**Authors:** Katarzyna Filipek, Łukasz Otulakowski, Katarzyna Jelonek, Alicja Utrata-Wesołek

**Affiliations:** Centre of Polymer and Carbon Materials, Polish Academy of Sciences, M. Curie-Skłodowskiej 34, 41-819 Zabrze, Poland

**Keywords:** poly[oligo(ethylene glycol) methacrylate]s, POEGMA, polymeric nanoparticles, thermoresponsive polymers, self-assembly, nanogel

## Abstract

Polymer nanogels—considered as nanoscale hydrogel particles—are attractive for biological and biomedical applications due to their unique physicochemical flexibility. However, the aggregation or accumulation of nanoparticles in the body or the occurrence of the body’s defense reactions still pose a research challenge. Here, we demonstrate the fabrication of degradable nanogels using thermoresponsive, cytocompatible poly[oligo(ethylene glycol) methacrylate]s-based copolymers (POEGMA). The combination of POEGMA’s beneficial properties (switchable affinity to water, nontoxicity, non-immunogenicity) along with the possibility of nanogel degradation constitute an important approach from a biological point of view. The copolymers of oligo(ethylene glycol) methacrylates were partially modified with short segments of degradable oligo(lactic acid) (OLA) terminated with the acrylate group. Under the influence of temperature, copolymers formed self-assembled nanoparticles, so-called mesoglobules, with sizes of 140–1000 nm. The thermoresponsive behavior of the obtained copolymers and the nanostructure sizes depended on the heating rate and the presence of salts in the aqueous media. The obtained mesoglobules were stabilized by chemical crosslinking via thiol-acrylate Michael addition, leading to nanogels that degraded over time in water, as indicated by the DLS, cryo-TEM, and AFM measurements. Combining these findings with the lack of toxicity of the obtained systems towards human fibroblasts indicates their application potential.

## 1. Introduction

Nanotechnology has gained interest in medicine for its potential to address challenges associated with conventional therapeutic agents. The use of nanoparticles (within the size range of 10 to 1000 nm [1]), with versatile structures and morphologies, allows us to overcome issues frequently associated with novel drugs, such as poor water solubility, lack of targeting capability, nonspecific distribution, and systemic toxicity [2,3]. Among nanoparticles, polymer-based ones (PNPs) are particularly promising. This interest arises from the fact that controlled polymerization processes can lead to polymers with complex microstructures, architectures, or functionalities, resulting in a wide range of physicochemical properties [4,5]. Moreover, many polymers are biologically inert and biodegradable, so these issues may mitigate concerns regarding their interactions within the body [6,7].

The careful choice of (co)polymer composition primarily determines the PNP physicochemical properties, morphology, functionality, and degradability. However, the sizes and shapes are also controlled by the choice of preparation technique and the environmental conditions used, such as polymer concentration, solvent composition, and the presence of additives [3]. PNPs can be fabricated through two major methods. The first one, the direct polymerization of monomers, employs techniques such as emulsion, mini-emulsion, dispersion, and precipitation polymerization [3]. This approach grants control over particle sizes through monomer concentration, surfactant selection, or reaction temperature [8]. The second involves the aggregation of pre-formed polymers to nanoparticles utilizing techniques such as nanoprecipitation, solvent evaporation, and coacervation [2]. When a crosslinking agent or polymers with defined crosslinkable functional groups are used during the process of nanoparticle formation, nanogels are formed. There are competent reviews, to which we refer the reader, where characteristics of the polymer nanoparticles, including nanogels, as well as morphological characterization and formation protocols are comprehensively described [1,2,3,6,8,9,10].

For the preparation of PNPs with unique properties, so-called smart polymers have proven to be very useful [11]. Such polymers reversibly change their physicochemical properties in response to external stimuli such as temperature [12], pH [13], light [14], and electromagnetic field [15]. Thermoresponsive polymers, for instance, are fully water-soluble at low temperatures. At higher temperatures, changes in the interaction between the polymer chain and water molecules cause a transition from a coiled to a globular chain conformation, leading to phase separation and aggregation, ultimately resulting in polymer precipitation [16]. When a dilute solution of thermoresponsive polymers is considered, upon heating above T_CP_, nanoparticles called mesoglobules are formed [17,18]. The exact temperature at which this process occurs is called the phase transition temperature (T_CP_).

Among the thermosensitive polymers, poly[oligo(ethylene glycol) methacrylate]s (POEGMAs) and their copolymers have recently garnered a lot of attention [18,19,20,21,22,23,24,25]. POEGMA have well-established bioinert and non-toxic properties, as no specific interactions of polymers with biological materials have been observed [26,27,28]. What is intriguing is that these polymers do not trigger an immune response, as they are not recognized by poly(ethylene glycol) (PEG) antibodies and do not cause the formation of anti-POEGMA antibodies [29,30,31]. This makes them promising replacements for PEG, which, to date, is the gold standard used in biomedicine [32]. POEGMAs are amphiphilic polymers thanks to the hydrophobic main chain and hydrophilic oligo(ethylene glycol) (OEG) side chains. When a proper balance between these parts is preserved and a certain OEG end group is used, they exhibit thermoresponsive behavior. The T_CP_ of POEGMAs in water can be controlled over a wide range of temperatures (20–90 °C) by altering the length of the OEG side chains and its end group [33] or by their copolymerization with other comonomers that can also contain reactive, functional groups [18,34,35]. The main advantage of the POEGMA thermoresponsiveness is the full reversibility of the phase transition in water, with no marked hysteresis [36].

The thermoresponsive behavior of POEGMA is mainly investigated in water, while determining the impact of dissolved additives, such as salts, surfactants, and amino acids, is mainly limited to reporting the changes in T_CP_ values. Studies show that the salts’ influence on the T_CP_ of POEGMA is in agreement with the Hofmeister series [37,38,39]. In turn, the T_CP_ values of POEGMA in culture media, e.g., DMEM, decrease as compared to those measured in water [37,40,41]. There are scarce studies of the effect of additives or heating rates on the POEGMA process of aggregation, particle formation, and stability in the vicinity of or above the T_CP_ [37,40]. It was observed that the presence of different salts affects not only the size, morphology, and stability of nanoparticles but also the aggregation process, leading, in some cases, to irreversible precipitation of the nanosystem, which does not dissolve even after cooling [40]. Such observations require detailed studies and are particularly important in the case of PNPs intended for medical and biological applications, because cell culture media and human body fluids contain large amounts of solutes.

POEGMAs are eagerly used to create nanoparticles with different structures, e.g., micelles, vesicles, worms, fiber-like or flower-like structures, hybrid nanoparticles, or nanogels (for example, [20,21,24,34,42,43,44]). In all these studies, the interest in this class of polymer encompasses its two functions: hydrophilicity, which ensures the bioinertness and anti-fouling properties [28,45], or thermoresponsivity, used for nanoparticle formation or drug encapsulation and release [46,47,48]. When considering nanogels of POEGMA, it should be noted that they are generally prepared using the precipitation polymerization methodology [22,49,50,51]. It combines two simultaneous processes, polymerization and crosslinking, in the presence of bifunctional agents such as ethylene glycol dimethacrylate, tetraethylene glycol dimethacrylate, or N,N’-methylene bis(acrylamide) [22,52,53]. Depending on the chemical composition and crosslinking degree, it is possible to tune nanogel properties such as size, swelling, or porosity. Nanogels with a heterogeneous structure may also be obtained, influencing their thermoresponsivity or mechanical properties, or the distribution of active substances [54,55]. A much less explored method for obtaining POEGMA nanogels involves crosslinking of the previously prepared polymer with complementary functional groups using the click reactions [18,21,48]. In this approach, under defined temperature, polymers form aggregates, and during highly selective and fast reaction, covalent bonds are created in aggregates’ interior, leading to stabilization. It was also described that the POEGMA nanogels may be prepared by crosslinking of the core or shell of the micelles [19,56] or during polymerization-induced self-assembly (PISA) with the presence of crosslinker [53].

In many cases, it is important that nanogels are degradable, enabling the polymer removal and/or the release of active substances. POEGMA itself is not degradable, although several solutions, such as the introduction of labile segments, derived from 2-methylene-1,3-dioxepane and its derivative [57,58,59], into the polymer backbone or formation of poly(lactic acid) degradable segments as grafts or blocks in the copolymer chain [27,60], were applied. In the case of POEGMA nanogels, degradable bonds were mainly derived from crosslinking agents such as disulfides (e.g., bis(2-acryloyloxyethyl) disulfide [61], N,N’-bis(acryloyl) cystamine) [62] or ketals (e.g., 2,2-bis(aminoethoxy)propane) [63] introduced into their structure during the synthesis via free radical copolymerization. In other described approaches, degradable hydrazone [21] and carbamate [64] bonds were formed within nanogels during the crosslinking of polymer precursors [18,21]. For example, Simpson et al. [21] mixed hydrazide and aldehyde functionalized POEGMA and analyzed the influence of different combinations of precursor polymer and reaction conditions (e.g., aggregation temperature, precursor concentration) on the formation of small (50 to 150 nm) and monodisperse nanogels. Lipowska-Kur et al. [18,64], in turn, obtained POEGMA-based nanogels via click crosslinking (Huisgen’s cycloaddition) of thermally co-aggregated mesoglobules of prepolymers with azide and propynyl carbamate groups. The resulting nanogel sizes were largely influenced by the polymer concentration, the heating rate, the number of functional groups, and thus, the crosslinking degree. This method was also applied to construct the degradable doxorubicin nanocarriers, opening up the possibility of their advanced applications.

Herein, we extend the approach to the formation of degradable POEGMA-based nanogels, achieved by thermally induced aggregation of polymer chains into mesoglobules followed by their chemical crosslinking. For this purpose, we obtained thermoresponsive copolymers of different oligo(ethylene glycol) methacrylates. Hydroxyl-terminated oligo(ethylene glycol) methacrylate (HOEGMA) was used as a comonomer, which enabled subsequent copolymer modification with degradable oligo(lactic acid) (OLA) segments. The OLA segments were then modified with acrylate groups by Steglich esterification reaction with acrylic acid. The behavior of copolymers in water, in aqueous salt solution, and in media used for the biological test was studied in order to assess their potential application as nanocarriers. The nanogels were prepared by tandem physical aggregation (due to the thermoresponsive properties of the copolymer) and chemical stabilization of the aggregates (via the Michael addition crosslinking reaction between acrylate groups of copolymer and thiolated crosslinking agents). Such methodology facilitated preparation of initially structurally stable POEGMA nanogels, with the possibility of their hydrolytic degradation via the hydrolysis of ester bonds in OLA segments. The polymers used for particle formation were evaluated for cytotoxicity against human fibroblast model cells, revealing their biomedical potential.

## 2. Materials and Methods

### 2.1. Materials

Ethyl 2-bromo-2-methylpropionate (EBiB, 98%), copper(I) chloride (CuCl, ≥98%), 2,2′-bipyridyl (Bpy, >99%), triethylamine (TEA, ≥99%), tin(II) 2-ethylhexanoate (Sn(Oct)_2_), N,N’-dicyclohexylcarbodiimide (DCC, 99%), 4-dimethylaminopyridine (DMAP, 99%), 2,2-(etylenedioxy) diethanethiol (EDDET, 99%), Dulbecco’s Modified Eagle’s Medium (DMEM), sodium chloride (NaCl, ≥95%), potassium chloride (KCl, ≥95%), and sodium carbonate (NaHCO_3_, ≥95%) were purchased from Sigma Aldrich (Steinheim, Germany) and used as received. Tri(ethylene glycol) monoethyl ether methacrylate (TEGMA_EE_, Mn = 246 g/mol) was purchased from ECEM European Chemical Marketing (Amsterdam, Holland) and used as received. Acrylic acid (AC, >99%, Fluka, Steinheim, Germany), acetone (99.9%, POCH, Gliwice, Poland), and methanol (MeOH) (99.9%, POCH, Gliwice, Poland) were used as received. The ion exchanger Sephadex LH-20 was purchased from GE Healthcare (Warszawa, Poland) and used as received. 

Oligo(ethylene glycol) methacrylate (OEGMA, Mn = 300 g/mol, Sigma Aldrich, Steinheim, Germany) was purified by passing over a short column of activated basic aluminum oxide to remove the inhibitor. Mono-hydroxy terminated oligo(ethylene glycol) methacrylate (HOEGMA, Mn = 360 g/mol, Sigma Aldrich, Steinheim, Germany) was purified by solvent extraction [65]. L-Lactide (98%, Sigma Aldrich, Steinheim, Germany) was sublimated under vacuum. 

Dichloromethane (DCM, 99.8%, POCH, Gliwice, Poland) was purified by distillation over CaH_2_ prior to use. Water was purified using a commercial ion exchange system (Hydrolab, Straszyn, Poland). Tetrahydrofuran (THF, 99.8%, POCH, Gliwice, Poland) was dried and distilled over KOH and then over Na/K (1/3) alloy. Dowex Marathon MSC ion exchanger was purchased from Sigma Aldrich (Steinheim, Germany) and transformed into H^+^ using 1.6 M HNO_3_. 

### 2.2. Polymer Synthesis

#### 2.2.1. Synthesis of Copolymers of Oligo(Ethylene Glycol) Methacrylates (POLY X_A_)

Polymerization conditions for the synthesis of copolymers of oligo(ethylene glycol) methacrylates were partially developed based on the existing literature [41]. The ratio of TEGMA_EE_ to OEGMA was changed, while a constant degree of polymerization (DP) of 10 for HOEGMA was kept. For the sake of clarity, the synthesis protocol is shown for a copolymer with a molar ratio of [TEGMA_EE_]/[OEGMA]/[HOEGMA] of 55/45/10. The atom transfer radical polymerization (ATRP) was performed using a catalytic system [EBiB]/[CuCl]/[Bpy] of 1/1/2. Firstly, the catalyst CuCl (30.05 mg, 3.04 × 10^−4^ mol) was dissolved in a mixture of methanol and water (13.41 mL, MeOH/H_2_O 2:1 *v*/*v*) in a Schlenk flask equipped with a magnetic stirrer and an argon/vacuum inlet valve solution, followed by the addition of the ligand (Bpy, 94.8 mg, 6.07 × 10^−4^ mol). After complete dissolution of Bpy, TEGMA_EE_ (4 mL, 1.66 × 10^−2^ mol), OEGMA (3.9 mL, 1.37 × 10^−2^ mol), and HOEGMA (1.04 mL, 3.03 × 10^−3^ mol) monomers were added to the flask. The final mixture was degassed by three freeze–vacuum–thaw cycles. Next, the initiator (EBiB, 44.56 μL, 3.04 × 10^−4^ mol) was added to the polymerization reaction mixture, and it was degassed once again. The reaction was carried out for 5 h at 25 °C. The obtained polymer solution was diluted in 15 mL THF and passed through DOWEX-MSC-1 ion-exchange resin to remove the copper catalyst. The obtained polymer was purified by dialysis in a membrane (SpectraPor membrane with MWCO 6000–8000 g/mol, Karlsruhe, Germany) against methanol and dried by lyophilization.

#### 2.2.2. Modification of Oligo(Ethylene Glycol) Methacrylate Copolymers with Oligo(Lactic Acid) Units (POLY X_B_)

An exemplifying procedure, similar to that described in the literature [66], is presented below. Copolymer POLY3_A_ (1g, 3.33 × 10^−4^ mol of -OH groups in copolymer) was added to a reactor, dried under vacuum, and dissolved in 3.6 mL of dry THF. After dissolution of a copolymer, L-Lactide (0.47 g, 3.33 × 10^−3^ mol) was added and the solution was stirred. The expected DP of L-lactide, regardless of the copolymer composition, was always 10. Then, 30.3 µL of Sn(Oct)_2_ (2.47 mol/L solution in dry THF) was transferred into the reactor under constant nitrogen flow. The polymerization was run at 55 °C for 14 days. The obtained polymer was purified by dialysis (MWCO 6000–8000 g/mol) for 2 days against acetone. After dialysis, the acetone was evaporated, and the polymer was dried to a constant weight under vacuum.

#### 2.2.3. Introduction of Acrylate Groups into Oligo(Ethylene Glycol) Methacrylate Copolymers (POLY X_C_)

An exemplifying procedure is presented for POLY3_B_. A total of 0.2g of the polymer (6.6 × 10^−6^ mol of -OH group in copolymer) was dissolved in 1 mL of DCM under a nitrogen atmosphere. Simultaneously, acrylic acid (1.02 × 10^−2^ mL, 1.5 × 10^−4^ mol) was complexed with DCC (3.4 × 10^−2^ g, 1.65 × 10^−4^ mol) in a separate reactor in 1 mL of DCM. The mixture was stirred under the nitrogen atmosphere for 1 h. Next, the contents of both reactors were mixed, and DMAP (1.8 × 10^−2^ g, 1.5 × 10^−4^ mol) was added under an argon atmosphere. The esterification was performed at 25 °C for 24 h. The resulting solution was filtered, and then an ion exchange agent (Sephadex) was added to remove the unreacted acrylic acid. The obtained polymer was purified by dialysis (MWCO 6000–8000 g/mol) for 2 days against acetone. After dialysis, the acetone was evaporated, and the polymer was dried to a constant weight under vacuum.

The obtained copolymers were designated as POLY X_A_, POLY X_B_, and POLY X_C_, where X represents the copolymer sample number; A designates the TEGMA_EE_, OEGMA, and HOEGMA copolymer, B designates the copolymer modified with OLA; and C designates the copolymer modified with OLA and the acrylate group.

### 2.3. Preparation of Nanogels

To obtain nanoparticles, two heating protocols, abrupt heating and nanoprecipitation, were applied. 

Abrupt heating: a total of 0.5 mg of copolymer was dissolved in 1 mL of H_2_O or aqueous solution of the appropriate salt (0.15 mol/L). Then, the EDDET crosslinker (97 µL, 0.97 g/L solution in H_2_O) and TEA (catalytic amounts) were added. The [SH]:[acrylate group] molar ratio was equal to 1:1. The prepared solution was abruptly heated to 70 °C by immersion in a preheated oil bath. 

Nanoprecipitation: in this method, the nanogels were formed according to the procedure described by N. Toncheva et al. [67]. The copolymer (0.5 mg), EDDET crosslinker (97 µL, 0.97 g/L solution in THF), and TEA (catalytic amounts) were dissolved in 1 mL of THF. Then, the prepared solution was added dropwise to 3 mL water or aqueous solution of the appropriate salt (0.15 mol/L) and preheated to 70 °C under vigorously stirring. Samples were kept at this temperature for 1 h to evaporate residual THF. 

For both heating protocols, all the samples were equilibrated at 70 °C for 2 h in order to perform simultaneous aggregation and crosslinking. After that time, the solutions were subjected to DLS measurements. 

### 2.4. Degradation of the Nanogels

The nanogels for the degradation studies were obtained via nanoprecipitation of the POLY3_C_ solution in THF in water at 70 °C. After 2 h of incubation, the nanogel suspension was cooled down to 40 °C, the temperature that imitates physiological conditions relevant to potential medical applications. The degradation process of the nanogels was monitored in detail using DLS, cryo-TEM, and AFM. The degradation process continued until the complete disappearance of nanoparticles, a crucial endpoint verified through comprehensive assessments using all three methods.

### 2.5. Methods

Gel Permeation Chromatography. The molar masses and molar mass distributions of the copolymer were appointed by gel permeation chromatography with multiangle laser light scattering detection (GPC-MALLS). The following column set was used: guard + GRAM 100 Å + GRAM 1000 Å + GRAM 3000 Å (Polymer Standards Service, PSS). Measurements were performed in DMF containing 5 mmol/L lithium bromide at 45 °C with a nominal flow rate of 1 mL/min. In the system, a differential refractive index detector (SEC-3010 WGE Dr. Bures, Berlin, Germany) and a multiangle laser light scattering detector (DAWN HELEOS from Wyatt Technologies, Santa Barbara, CA, USA, λ = 663.8 nm) were used. ASTRA 7 software ver. 7.3.1.9 (Wyatt Technologies, Santa Barbara, CA, USA) was used to evaluate the results. The value of the refractive index increment (dn/dc) was determined on a SEC-3010 dn/dc refractometer detector with a wavelength of λ = 620 nm. Measurements were made for 5 solutions with different concentrations in the defined range of 0.5–10 g/L at 45 °C. For each concentration, at least three measurements were made. Data recording and calculations were performed using BI-DNDCW software ver. 5.31. The refractive index increment of copolymers was independently measured in DMF and was equal to 0.05.

Nuclear magnetic resonance spectroscopy. The ^1^H NMR spectra of copolymers were recorded on a Bruker Ultrashield spectrometer (Bruker, Billerica, MA, USA) operating at 600 MHz using CDCl_3_ as the solvent.

Cloud Point Measurements. The cloud points were measured using a Specord 200plus UV-Vis spectrophotometer (Analytik Jena, Jena, Germany). The transmittance was monitored as a function of temperature at a wavelength λ = 500 nm with constant stirring. Transmittance values were recorded every 1 °C after 60 s stabilization. The concentration of the polymer solution was 1 g/L. The T_CP_ value was determined as the temperature at which the transmittance of the polymer solution reached 50% of transmittance drop. The T_CP_ of the copolymers was measured in water, DMEM, and in different salt solutions, namely NaCl, KCl, and NaHCO_3_ (c = 0.15 mol/L). 

Dynamic light scattering (DLS) was used to determine the sizes and size distributions of particles in water. Measurements were performed using a Brookhaven BI-200 (Brookhaven Instruments, New York, NY, USA) goniometer with a vertically polarized laser light (Brookhaven Instruments, New York, NY, USA) of wavelength λ = 637 nm (semiconductor laser diode 36 mW) and equipped with a Brookhaven BI-9000 AT digital autocorrelator. The intensity of scattered light was measured at an angle of 90° at various temperatures. The autocorrelation functions were analyzed using the constrained regularized CONTIN method to obtain distributions of relaxation rates (Γ). The latter provided distributions of the apparent diffusion coefficient D (D = Γ/q_2_, where q is the magnitude of the scattering vector, q = 4πn/λ·sinθ/2, and n is the refractive index of the medium). The apparent hydrodynamic diameter (D_h_^90^) was obtained from the Stokes–Einstein equation (Equation (1)).

D_h_^90^ = kT/6πηD (1)

for θ = 90°, where k is the Boltzmann constant and η is the viscosity of water at temperature T. The sizes of the nanogels were measured at 70 °C following synthesis and at 40 °C during degradation.

Cryogenic transmission electron microscopy (cryo-TEM). Cryo-TEM images were obtained using a Tecnai F20 X TWIN microscope (FEI Company, Hillsboro, OR, USA) equipped with a field emission gun, operating at an acceleration voltage of 200 kV. Images were recorded on the Gatan Rio 16 CMOS 4k camera (Gatan Inc., Pleasanton, CA, USA) and processed with Gatan Microscopy Suite (GMS) software ver. 3.31.2360.0 (Gatan Inc., Pleasanton, CA, USA). Specimen preparation was performed by vitrification of the aqueous solutions on grids with holey carbon film (Quantifoil R 2/2; Quantifoil Micro Tools GmbH, Großlöbichau, Germany). Prior to use, the grids were activated for 15 s in oxygen plasma using a Femto plasma cleaner (Diener Electronic, Ebhausen, Germany). Cryo-samples were prepared by applying a droplet (3 μL) of the suspension to the grid, blotting with filter paper, and immediately freezing in liquid ethane, using a fully automated blotting device, Vitrobot Mark IV (Thermo Fisher Scientific, Waltham, MA, USA). After preparation, the vitrified specimens were kept under liquid nitrogen until they were inserted into a cryo-TEM-holder Gatan 626 (Gatan Inc., Pleasanton, CA, USA) and analyzed in the TEM at −178 °C.

Atomic Force Microscopy (AFM). Samples in water solution were dropped on a mica disc slide and spin-coated (SPIN150, SPS-Europe B.V., Putten, Netherlands) over 1 h with a rotation speed of 400 rpm and dried for 24 h. AFM images were obtained using a MultiMode with a Nanoscope IIId controller, Veeco (New York, NY, USA). The measurements were carried out in air, with a nominal scan range of 10 × 10 µm^2^. The imaging of samples was conducted in the tapping mode at a scan rate of 1 Hz using etched silicon probes (PPP-NCH-10, NANOSENSORS) of nominal spring constant 42 N/m and operating at a resonant frequency of 320 kHz. Micrographs were recorded using NanoScope Software V531r1.

Cytotoxicity Assay. The in vitro cytotoxicity study was conducted according to the ISO 10993-5 standard [68]. The human fibroblasts WI-38 (CCL-75) were obtained from the ATCC cell bank. The cells were cultured in Dulbecco’s Modified Eagle’s Medium—high glucose (DMEM) supplemented with 10% fetal bovine serum, 100 U/mL penicillin, and 100 μg/mL streptomycin. The cells were cultivated at 37 °C, in a humidified atmosphere containing 5% CO_2_. To study cytotoxicity, 100 μL of cell suspension, containing 6 × 10^3^ cells, was transferred to wells of the 96-well plates and cultured in standard medium for 24 h to ensure cell adhesion. After 24 h, the medium was exchanged with the medium containing the tested material. The tested material was prepared directly before the experiment by dissolving the POLY 3_C_ polymer in DMEM at the concentration of 10 mg/mL, filtering through 0.2 µm syringe filters, and then diluting in the range of 0.001–10 mg/mL. The cells were incubated with the tested samples for 24–72 h. Untreated cells were used as negative controls, and cells treated with 5% of DMSO as positive control. The viability of cells was evaluated with the use of Cell Counting Kit-8 (CCK-8). Absorbance was read at 450 nm (reference: 650 nm) at the Spark 10M (Tecan, Männedorf, Switzerland). The results were analyzed using a one-way ANOVA followed by a Tukey post hoc test. A *p* value of <0.05 was considered statistically significant.

## 3. Results

### 3.1. Synthesis and Characterization of Poly[Oligo(Ethylene Glycol)Methacrylate] Derivatives

**Figure 1:**
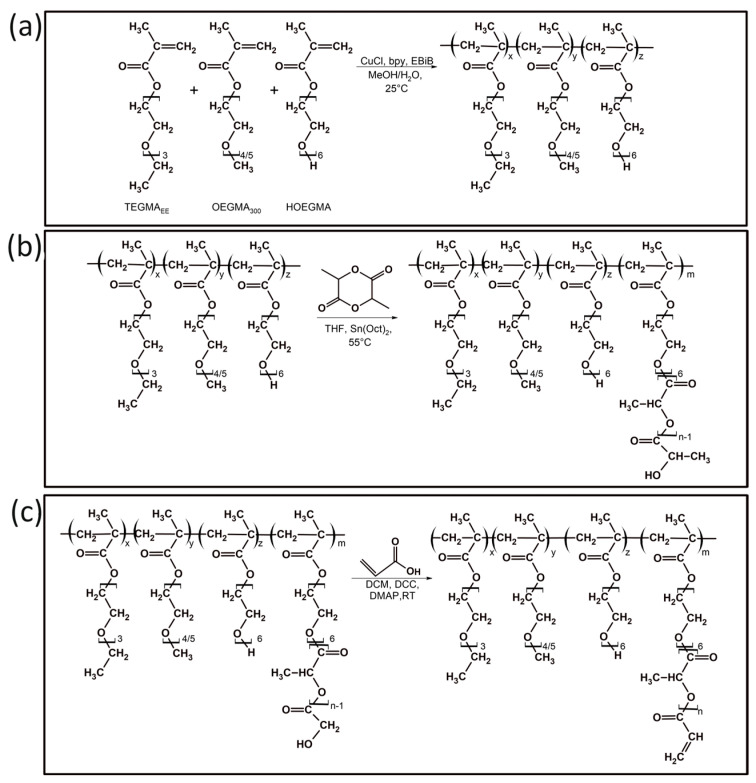
Synthesis of POEGMA-based copolymers: (**a**) the ATRP of oligo(ethylene glycol) methacrylates; (**b**) the ROP of L,L-lactide initiated by hydroxyl groups of the polymer; (**c**) modification of copolymer with acrylic acid.

The synthesis of poly[oligo(ethylene glycol)methacrylate] derivatives included three steps. First, the atom transfer radical polymerization (ATRP) of oligo(ethylene glycol) methacrylates (TEGMA_EE_, OEGMA_300_ and HOEGMA) was performed (Figure 1a). In the next step, the hydroxyl group at the end of HOEGMA side chains acted as an initiator of ring opening polymerization (ROP) of L,L-lactide catalyzed by Sn(Oct)_2_, enabling the copolymer modification (Figure 1b). Finally, the hydroxyl group at the end of the oligo(lactic acid) side chains was substituted with acrylic acid in a Steglich esterification reaction (Figure 1c). The esterification process of hydroxyl groups of HOEGMA not substituted with OLA was not taken into account. As we further present, the obtained nanogels undergo degradation, which is possible only via the hydrolysis of OLA units in the crosslinked HOEGMA-OLA segments. If esterification occurred on the remaining HOEGMA hydroxyl groups, the subsequent crosslinking of the polymer nanoparticles would not result in degradable nanogels. This approach to the synthesis of copolymers was intended to enable crosslinking of the nanostructures, formed by the copolymer, during the Michael addition reaction between acrylate groups and the crosslinker equipped with two thiol groups. The presence of oligo(lactic acid) units was expected to promote, at a later stage, the degradation of the obtained nanogels.

The ATRP of oligo(ethylene glycol) methacrylates yielded copolymers with a molar mass of around 40 000 g/mol, as measured by GPC-MALLS. The GPC signal of all copolymers, and that after modification with L,L-lactide and acrylic acid, is symmetrical and monomodal, and the copolymer dispersity (Ð) is about 1.5 (Figure S1). 

**Figure 2:**
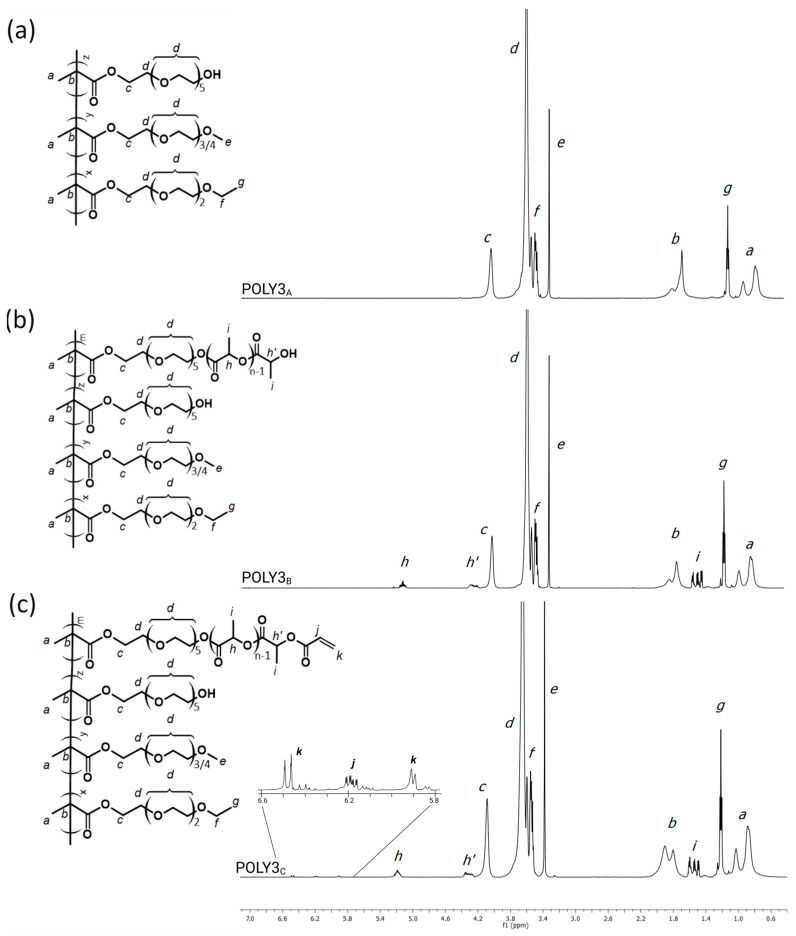
^1^H NMR spectra for representative POLY3 series (CDCl_3_): (**a**) TEGMA_EE_:OEGMA_300_:HOEGMA copolymer; (**b**) copolymer modified with OLA segment: (**c**) copolymer containing OLA segment ending with acrylate groups.

The composition of the received copolymers was quantified based on ^1^H NMR spectra (Figure 2), and it was confirmed that well-defined copolymers with a different comonomer ratio were obtained (Table 1). For calculation, the ratio of peak integration at a chemical shift of 1.2 ppm (peak g), attributed to the protons of the methoxy group from the TEGMA_EE_ side chain, to peak integration at a chemical shift of 3.4 ppm (peak e), attributed to the methoxy group in the OEGMA side chain, was taken into account (Figure 2a). The content of the HOEGMA in the copolymers was 8–9%mol, which was determined from the difference in the peak integration between the signals belonging to protons of the methylene group of all comonomers (peak c) and those of the methoxy group from the TEGMA_EE_ (peak g) and OEGMA_300_ (peak e). It occurred that the composition of the TEGMA_EE_–OEGMA_300_–HOEGMA copolymers (series “A” in Table 1) is in relatively good agreement with the targeted composition. 

The use of ROP allowed for the attachment of the oligo(lactic acid) segments into the copoly[oligo(ethylene glycol) methacrylate] side chains (Table 1, polymers denoted as series “B”). In the ^1^H NMR spectra (Figure 2b), signals at δ = 1.6 ppm (peaks i) correspond to the protons of the methyl group of the OLA chain, whereas signals at δ = 4.4 ppm (peaks h’) and δ = 5.25 ppm (peaks h) are attributed to the terminal methine proton and methine proton of the OLA segment, respectively. The presence of the aforementioned signals strongly supports the successful modification of the POEGMA copolymers with L,L-lactide. The number of HOEGMA hydroxyl groups substituted with the OLA segment was calculated by comparing the peak integrations of the methylene group (peak c) before and after L,L-lactide modification. The length of OLA was calculated from the ratio of peak integration of the substituted HOEGMA hydroxyl group with L,L-lactide units (peak c) to the peak integration of the methyl group of the OLA chain (peak i). About 45% of hydroxyl groups in initial copolymers were modified with OLA segments of DP ranging from 6 to 12 (Table 1).

The introduction of a double bond, derived from acrylic acid, into the side chain of the OLA-modified copolymers was performed using an esterification reaction (Table 1, polymers denoted as series “C”). Acrylic acid was used instead of methacrylic acid due to its higher reactivity in the thiol-Michael addition reaction, which is used in the next step to prepare nanogels [69]. The presence of the characteristic signals derived from the protons of acrylate groups (5.8, 6.15, and 6.5 ppm, peaks j and k) confirmed the successful modification of the copolymers with acrylic acid (Figure 2c). The acrylation degrees of all copolymers were calculated based on comparison of the peak integrations of the methylene group (peak c) of the HOEGMA and the signal derived from the protons of the double bond (peak j) (Table 1).

### 3.2. The Thermoresponsive Behavior of Copolymers 

The thermoresponsive behavior of POEGMA-based copolymers synthesized in this work was investigated in water, in aqueous salt solutions, and in Dulbecco’s modified Eagle’s medium (DMEM). The studies of thermoresponsive behavior of the obtained copolymers under conditions closely resembling those of physiological and cell culture environments are of key importance, if their biological applications are considered, as unexpected aggregation processes may occur [40]. 

**Figure 3:**
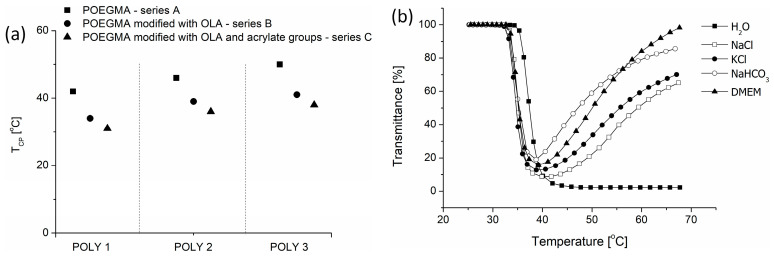
(**a**) Cloud point temperatures for POEGMA-modified copolymers in water (1 g/L); (**b**) transmittance as a function of temperature (500 nm, 1 °C/min), measured in different salt solutions for POLY3_C_, as a representative copolymer.

All synthesized copolymers exhibit thermoresponsive behavior in all solutions. Their T_CP_ ranged from 30 °C to 50 °C (Figure 3a), depending on the copolymer composition and the type of group introduced into the chain; also, the type of solvent used influenced the transition temperature value (Figure 3b).

It is known that the T_CP_ of POEGMA copolymers can be altered by changing the content of hydrophilic and hydrophobic segments in the copolymer chain [70]. Here, as the amount of the most hydrophilic HOEGMA was kept at a similar level for all copolymers, by increasing the content of other hydrophilic comonomer OEGMA_300_, it was possible to increase the T_CP_ value of the resultant copolymers from 42 °C to 50 °C (Figure 3a—squares). The subsequent modification of the POEGMA-based copolymer and the introduction of OLA segments and the acrylate group in the side chains resulted in a gradual decrease of T_CP_ in comparison to the unmodified copolymer (Figure 3a—circles and triangles). The introduction of the hydrophobic OLA segments (between 6 and 12 mers per HOEGMA comonomer) caused a decrease in T_CP_ of about 7–9 °C in comparison to the unmodified polymer. A subsequent acrylation of OLA-modified POEGMA copolymers led to a further decrease of T_CP_ by an additional 2–4 °C. The alterations in temperature were analogous across all polymer series (POLY1, POLY2, and POLY3) following each modification. The obtained results strongly suggest that the chosen synthetic strategy is consistent, regardless of copolymer composition.

The thermoresponsive behavior for all POEGMA copolymers in water was completely reversible, with an absence of hysteresis (Figure S2). Above the phase transition temperature in water, copolymers formed aggregates that were stable and did not precipitate from the solution, even if the solution was heated to high temperatures. This feature applies both for the initial copolymer and its modified variants (those substituted with OLA and acrylate groups). The consistent and repeatable thermal response highlights the strong reversibility of the POEGMA–copolymer system in water, even after a variety of chemical modifications. 

The next stage of the research was to examine the behavior of copolymers in aqueous solutions containing additives. The thermoresponsive behavior studies in Dulbecco’s modified Eagle’s medium (DMEM) and in aqueous salt solutions (Figure 3b) were solely performed for POEGMA copolymers modified with OLA and acrylates (copolymers of series C), as they were used for nanogel formation. It was observed that the T_CP_ of copolymers decreased in the presence of salts and DMEM by about 3 °C in comparison to measurement performed in water. This is due to the fact that kosmotropic salts, e.g., NaCl, NaHCO_3_, and KCl, also present in the DMEM, cause a “salting out” effect [40]. In the presence of these salts, the hydration sphere of the copolymer chains changes, and the interactions between macromolecules and water are reduced. Consequently, this results in reduced solubility of the polymer during temperature increases.

In contrast to pure water, a significant difference between the heating and cooling cycles was observed in salt solutions and in DMEM for all tested copolymers (Figure S3). The observed phase transition was not fully reversible. The heating of the thermoresponsive copolymer in DMEM and salt solutions led to the decrease of transmittance due to the polymer thermoresponsive transition. However, at certain temperatures, the transmittance reached a minimum, and upon further heating above the transition temperature, it started to increase again. This was caused by the secondary aggregation and macroscopic precipitation of the polymer aggregates. The formed precipitate was exceptionally stable and did not dissolve during cooling of the sample. We observed that part of the precipitate dissolved only after prolonged storage in the fridge. Similar behavior was observed for several thermoresponsive polymers, as well as those based on copolymers of OEGMA with hydroxyethyl methacrylate, tested in phosphate buffer (PBS) and solutions of its component salts (NaH_2_PO_3_, Na_2_HPO_4_, NaCl) [37,71,72]. The heating of these polymers in salt solutions above the T_CP_ led to polymer precipitation, and the resulting precipitate remained stable during cooling. It was observed that the type of salt ion, its concentration, and the polymer chain’s structure significantly affected the aggregation process, stability, and susceptibility to irreversible precipitation.

### 3.3. Preparation of Mesoglobules and Their Crosslinking to Nanogels

The literature describes various methods of obtaining mesoglobules from thermoresponsive polymers, such as slow and abrupt heating or nanoprecipitation [17,67,73]. Each of these techniques has its pros and cons and influences the size and morphology of the particles. Generally, slow/gradual heating induces the formation of larger mesoglobules with broader size distribution, in comparison to the abrupt heating that commonly results in small particles with uniform sizes. This distinction arises from the observation that fast heating promotes more pronounced intrachain contraction and reduces interchain association [74]. The type of heating rate also affects the structure of the mesoglobules formed by the mixture of thermoresponsive polymers, leading to core-shell particles when gradual heating is applied [64,75]. In turn, nanoprecipitation led to the smallest particles composed of uniformly mixed polymer chains with good reproducibility, as compared to other heating methods [67,73]. 

**Figure 4:**
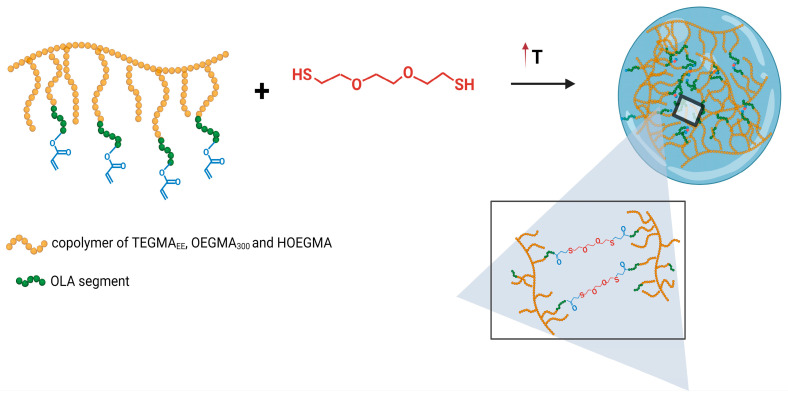
Simplified overview of the preparation of POEGMA-based nanogel via thermally induced aggregation of polymer chains followed by their chemical crosslinking.

For the purpose of this work, in order to obtain POEGMA-based nanogels, nanoprecipitation and abrupt heating were applied. The thermoresponsivity of the copolymers was employed to create mesoglobules. During their formation, encapsulation of the crosslinking agent was possible, which enabled a subsequent crosslinking via the Michael addition reaction between acrylate groups present in POEGMA side chains and thiol groups of the crosslinking agent (Figure 4).

**Figure 5:**
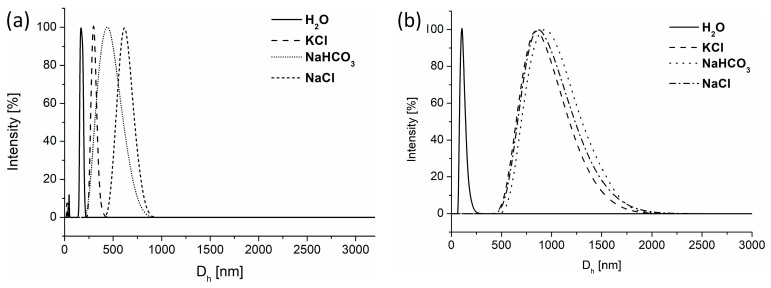
Size distribution of POLY3_C_ nanoparticles obtained via (**a**) nanoprecipitation and (**b**) abrupt heating, measured at 70 °C.

In the first step, the sizes of aggregates formed by the POLY3_C_ copolymer in abrupt and nanoprecipitation processes were analyzed via DLS in water and salt solutions (Figure 5, Table 2). In the case of abrupt heating, the vial containing the polymer and crosslinker in water or in salt solution was quickly immersed in a bath preheated to 70 °C. Nanoprecipitation, in turn, involved the dropwise addition of a polymer and crosslinker solution in THF into water or an aqueous salt solution preheated to 70 °C. This temperature significantly surpasses the cloud point of POLY3_C_, which is helpful in reducing the sizes of particles [17].

**Figure 6:**
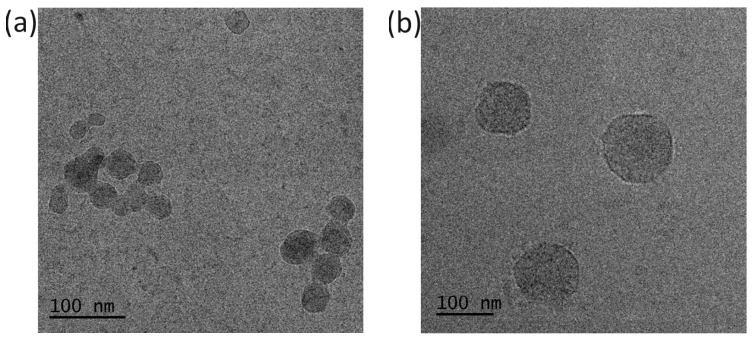
Cryogenic transmission electron microscopy (cryo-TEM) images of nanoparticles formed by POLY3_C_ obtained by (**a**) nanoprecipitation and (**b**) abrupt heating at 70 °C.

The hydrodynamic diameters of the obtained structures were in the range of 140 nm to 1000 nm, indicating a significant impact of the heating protocol and the presence of salt on their sizes (Table 2). The hydrodynamic diameters of the structures obtained in water were noticeably the smallest, and interestingly, no significant differences in sizes detected by DLS were observed, taking into account the heating protocol. Further investigation of these samples by cryo-TEM revealed that the morphology of formed aggregates in water differs. A nanoprecipitation fraction of 165 nm, observed by DLS, is composed of smaller particles of sizes of around 50 nm merged together (Figure 6a). In an abruptly heated sample, the uniform particles (Figure 6b), with sizes corresponding to those obtained by DLS, are present, indicating that the heating protocol had a prominent impact on particle size and morphology.

An utterly different situation occurs when nanoparticles are created in aqueous KCl, NaHCO_3_, or NaCl solutions. Firstly, their sizes are much larger than those obtained in water regardless of the method used. As a result of the “salting out” effect, not only was the thermoresponsive behavior of the polymer changed (Figure 3b and Figure S3), but the size of the resulting structure increased (Figure 5). Salts generate changes in the hydration sphere of copolymer chains, influencing their interactions with water molecules and, consequently, reducing the solubility of the polymer, inducing its aggregation into bigger structures [37,40]. Secondly, the effect of the heating protocol on the sizes is observed. Nanoprecipitation of the copolymer into a preheated aqueous salt solution, depending on the type of salt, resulted in particles of sizes ranging from 270 nm to 620 nm. These sizes were from almost two to nearly four times smaller than in the case of nanoparticles obtained by abrupt heating in the same salt solutions. In nanoprecipitation, the diffusion of the polymer into the aqueous phase is more rapid and spontaneous, leading to the immediate formation of nanostructures. This process aims to avoid water molecules, which can be enhanced by salts, and at this temperature, such solution acts as a precipitant for the polymer. This quick process greatly reduces the time needed for chain entanglement in comparison to abrupt heating. In abrupt heating, reaching transition temperature is significantly longer than in nanoprecipitation. This gives polymer chains more time to interact with each other, and salt presence modifies intermolecular interactions, affecting agglomeration kinetics with salt molecules, which lead to the appearance of bigger particles. Finally, in the nanoprecipitation protocol, an influence of the type of salt on the sizes can also be observed. Nanogels of the largest sizes were created in NaCl solution. This effect was not observed when abrupt heating was applied. 

**Figure 7:**
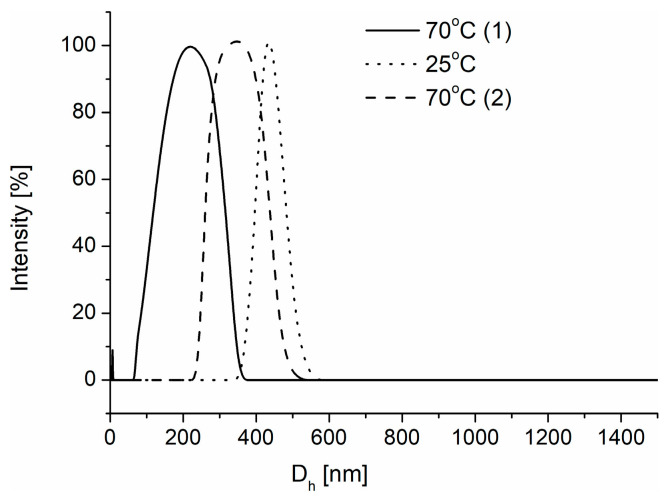
Size distribution of POLY3_C_ nanoparticles obtained by nanoprecipitation during the heating–cooling cycles.

Since a crosslinking agent was used during the creation of nanoparticles (Figure 4), in the next stage of research, the effectiveness of crosslinking reaction and the formation of nanogels for both heating protocols was verified. For this purpose, the nanoparticles, after their formation, were subjected to heating–cooling cycles. After synthesis at 70 °C, the nanoparticles were cooled to 25 °C. The sizes of nanoparticles formed via nanoprecipitation in water increased to 450 nm (Figure 7). At this temperature, which is below the T_CP_ of the polymer (Table 1), the nanoparticles became hydrophilic, and because the polymer chains were crosslinked, they absorbed water, leading to swelling. The dissolution of nanoparticles was not observed. Reheating the nanogels to 70 °C, that is, above the T_CP_ of polymer, resulted in their shrinking, and their size was reduced. A nanoparticle obtained via abrupt heating, despite using the crosslinking agent, did not form stable nanogels. After cooling, no nanoparticles were detectable. In DLS measurements, the light scattering intensity decreased to extremely low levels, confirming the absence of particles in the solution.

### 3.4. Degradation of Nanogels

The emphasis on developing surfactant-free synthesis of POEGMA nanogels of appropriate size and additional degradability represents a critical yet underexplored route, holding potential for future research. We have shown that such requirements can be met by the aggregation of thermoresponsive polymer chains into mesoglobules, using a rapid heating protocol like nanoprecipitation, resulting in nanometer-sized aggregates and further stabilized by crosslinking through click chemistry. The possibility of degradation of POEGMA nanogels is ensured by the presence of hydrolytically degradable OLA segments within their structure.

**Figure 8:**
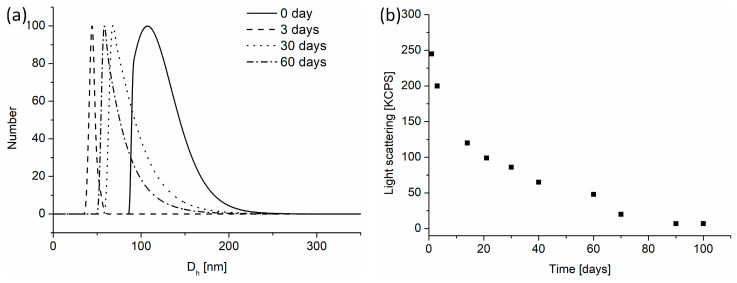
The degradation of POLY3_C_ nanogels monitored by DLS at 40 °C: (**a**) size distribution changes and (**b**) changes in light scattering.

**Figure 9:**
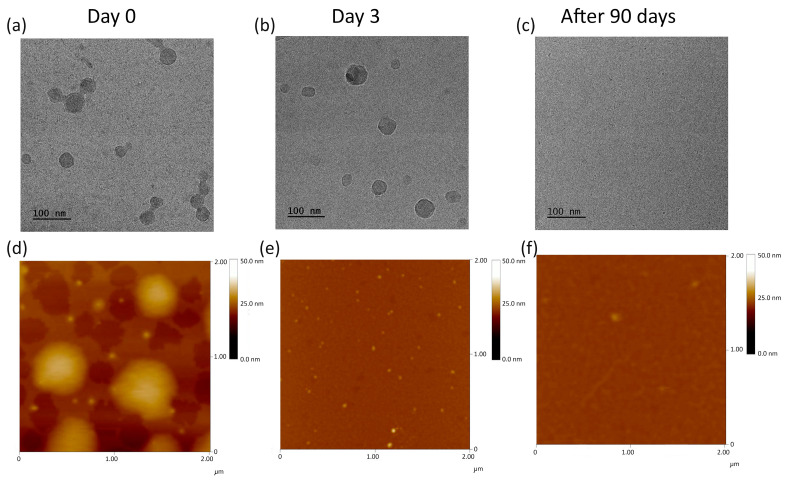
Cryogenic transmission electron microscopy (cryo-TEM) (**a**–**c**) and atomic force microscopy (AFM) (**d**–**f**) images during degradation process of POLY3_C_ nanogels.

The degradation process was followed for nanogels obtained via nanoprecipitation of the POLY3_C_ copolymer into water preheated to 70 °C. Then, the solution was cooled to 40 °C, and the degradation process was analyzed at various time intervals, with measurements conducted using DLS (Figure 8), cryo-TEM, and AFM (Figure 9). A temperature of 40 °C is close to physiological conditions but still exceeds the phase transition of the copolymer (Table 1), ensuring that the particles are in a shrunken state. After lowering the temperature from 70 °C to 40 °C, based on DLS measurements, no significant changes in the size of the nanogels were observed, showing particles of sizes of 160 nm (Figure 8a). The microscopic observation revealed that at that temperature, similar to particles obtained at 70 °C (Figure 6), a large number of particles were composed of a few merged-together nanogels of small size (around 50 nm) (Figure 9a,d).

A subsequent measurement after 3 days showed the beginning of degradation, which led first to disaggregation of larger aggregates into individual nanogels (Figure 9b,e). This was accompanied by a decrease in light scattering, with the main particle fraction detected by DLS being around 50 nm. The degradation process was prolonged and monitored by measurements of light scattering intensity (Figure 8). During the degradation within 60 days, the sizes of nanogels increased slightly, and their size distribution was broader (Figure 8a). Simultaneously, the scattering intensity gradually dropped, suggesting that the number of nanoparticles present in solution decreased. This means that the nanogels were disintegrating. After 90 days of degradation, the light scattering reached extremely low levels, signifying the absence of particles in the solution and indicating the completion of the degradation process. The findings were confirmed by cryo-TEM and AFM analyses. Despite in-depth and careful analysis of samples, no nanogels were identified via cryo-TEM and AFM (Figure 9c,f).

There are two simultaneous mechanisms involved in the destabilization process of POEGMA-based nanogels. The first is connected to the hydrolysis of the non-acrylated OLA side chains in the copolymer, as it was estimated that in POLY3_C_ nanogels, 17% of OLA hydroxyl groups were modified with acrylic acid (Table 1, Figure 4). The second one concerns the chain scission of the lactic acid esters present in the crosslinking points. As it was observed for N-(2-hydroxypropyl) methacrylamide esterified with oligo(lactic acid)s, the degradation of the hydroxyl-terminated oligo(lactic acid) segments proceeds much faster than the oligomers with the protected chain end (degradation performed in physiological conditions 37.8 °C, pH 7.2) [76,77]. The increase in nanogel size observed in these studies after several days of degradation is caused by the scission of the side OLA groups (both non-acrylated and those present in the crosslinking points), which caused an increase in the hydrophilicity of the polymer. This effect led to swelling of the particles (both due to the increased hydrophilicity and lowered crosslinking degree), which manifested itself in particles with larger sizes.

Taking into account the biological applications of the obtained nanogels, the issue of degradation certainly requires more detailed research. It is necessary to establish conditions that can accelerate the hydrolysis of ester bonds and thus nanogel destabilization, as well as to investigate the degradation of particles in biologically relevant media, which is the subject of the impending publication.

### 3.5. The Evaluation of the Cytotoxicity

**Figure 10:**
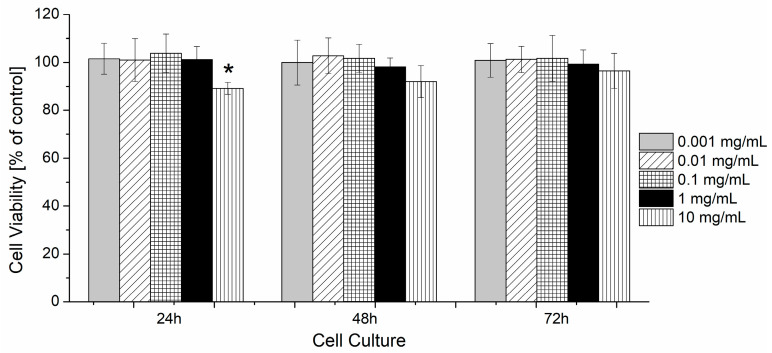
Cytotoxicity assay of POLY3_C_ at increasing concentrations (given in mg/mL). The assay was performed with human fibroblasts WI-38. The results are shown as a percentage of the control, where untreated cells constituted 100%. *p*-value of statistical significance is represented as * *p* < 0.05.

The in vitro cytotoxicity of the tested POEGMA-based material (POLY3_C_) was evaluated with the use of CCK-8 assay. The CCK-8 is a sensitive colorimetric technique for the determination of the number of viable cells using WST-8 (2-(2-methoxy-4-nitrophenyl)-3-(4-nitrophenyl)-5-(2,4-disulfophenyl)-2H tetrazolium, monosodium salt), which is reduced by cellular dehydrogenases to an orange formazan product. The amount of the produced formazan is directly proportional to the number of living cells. The results presented in Figure 10 show that the viability of cells was not affected by the presence of the tested material in the concentration range of 0.001–1 mg/mL after 24, 48, and 72 h. The highest concentration of the tested compound (10 mg/mL) caused a miniscule decrease of cell viability, by around 10%. However, this effect was statistically significant only for cells exhibiting POLY 3_C_ for 24 h. After longer times (48 and 72 h), the cell growth increased to 92 and 96%, respectively. Therefore, the POLYC_3_ copolymer was cytocompatible and thus potentially useful for more in-depth biological testing. 

## 4. Conclusions

Herein, we showed the possibility of preparing degradable POEGMA-based nanogels by thermally induced aggregation of polymer chains into mesoglobules followed by their chemical crosslinking. For this purpose, copolymers of different oligo(ethylene glycol) methacrylates were obtained by ATRP and subjected to further modification by ROP and esterification reactions, introducing acrylated-oligo(lactic acid) segments into the copolymer chain. It was estimated that the thermoresponsive behavior of copolymers may by controlled by the copolymer composition or by the presence of additives. A copolymer with a T_CP_ value close to the temperature of the human body may be obtained, which gives great opportunities for potential applications in tissue engineering or controlled drug delivery. It was shown that the thermoresponsive behavior of the copolymers in DMEM and salt solutions is different than in water. The presence of salt also induced macroscopic precipitation of the polymer aggregates. Crucially, the observed precipitation above the phase transition was not fully reversible. 

During abrupt heating or nanoprecipitation, the obtained copolymers undergo self-assembling and form nanoparticles with sizes of 140–1000 nm, depending on the heating rate and the presence of salts in the aqueous media. The subsequent crosslinking reaction by Michael addition (between acrylate groups of the copolymer and thiolated crosslinking agents) leads to the chemical stabilization of the aggregates. Only the nanoparticles obtained via nanoprecipitation were effectively chemically crosslinked, leading to nanogels.

The preliminary destabilization test confirmed the possibility of hydrolytic degradation of POEGMA-based nanogels. Furthermore, the cytotoxicity assay proved that the obtained material is nontoxic to fibroblasts over a wide range of concentrations. These findings, along with the knowledge about the behavior of nanogels in biologically relevant media, are important and may serve as a starting point for further research related to the preparation of POEGMA-based nanogels for encapsulation of bioactive substances and their use as drug delivery systems.

## Figures and Tables

**Table 1:** Characteristics of obtained copolymers.

	A—POEGMA Copolymer					B—POEGMA Substituted with OLA			C—POEGMA Substituted with OLA and Acrylic Acid	
	TEGMA_EE_/ OEGMA_300_/ HOEGMA ^1^	M_n theoretical_ [g/mol]	TEGMA_EE_/ OEGMA_300_/ HOEGMA ^2^	M_n_ [g/mol] and Ð ^3^	T_CP_ [°C] ^4^	Lactide/OH [mol %] ^2^	DP_OLA_^2^	T_CP_ [°C] ^4^	Acrylic Bond/OH [mol %] ^1^	T_CP_ [°C] ^4^
POLY1	75/25/10	29 600	69/22/8	37,000 1.49	42	45	12	34	47	31
POLY2	65/35/10	30 000	63/32/8	40,000 1.44	46	45	6	39	20	36
POLY3	55/45/10	30 600	54/41/9	41,000 1.50	50	44	9	41	17	38

^1^ Molar ratio of comonomers in the polymerization mixture. ^2^ Copolymer composition calculated from ^1^H NMR spectrum. ^3^ Measured by GPC-MALLS in DMF. ^4^ Determined by UV-Vis.

**Table 2:** Hydrodynamic diameter (D_h_) of nanoparticles formed by POLY3_C_ obtained by nanoprecipitation and abrupt heating (measurements performed at 70 °C).

Solvent	Nanoprecipitation	Abrupt Heating
	D_h_ [nm]	D_h_ [nm]
H_2_O	165	140
KCl	270	980
NaHCO_3_	450	1000
NaCl	620	980

## Data Availability

Data are contained within the article and Supplementary Materials.

## Supplementary Materials

The following supporting information can be downloaded at: https://www.mdpi.com/article/10.3390/polym16081163/s1, Figure S1: GPC-MALLS chromatograms for a representative poly[oligo(ethylene glycol) methacrylate] (POLY 3_A_), its derivative containing OLA (POLY 3_B_) and derivative containing OLA ended with acrylate groups (POLY 3_C_)).; Figure S2: The heating–cooling cycles for (a) POLY 3_A_, (b) POLY 3_B_, (c) POLY 3_C_ solutions in water (1 mg/mL).; Figure S3: The heating-cooling cycles for (a) various POEGMA copolymers modified with OLA and acrylates in DMEM; (b) POLY 3_C_ copolymer in different salts.
