# Supplementary material for: Degradable Nanogels Based on Poly[Oligo(Ethylene Glycol) Methacrylate] (POEGMA) Derivatives through Thermo-Induced Aggregation of Polymer Chain and Subsequent Chemical Crosslinking
Source: Polymers (Basel). 2024 Apr 20;16(8):1163. doi: 10.3390/polym16081163 (PMC11054481; doi:10.3390/polym16081163)
Supplement: Supplementary file 1 [file polymers-16-01163-s001.zip › polymers-2931031-supplementary.pdf]

SUPPORTING INFORMATIONS

Degradable nanogels based on POEGMA derivatives through thermo-induced aggregation of polymer chain and subsequent chemical crosslinking

Katarzyna Filipek, Łukasz Otulakowski, Katarzyna Jelonek, Alicja Utrata-Wesołek*

Centre of Polymer and Carbon Materials, Polish Academy of Sciences, M. Curie-Skłodowskiej 34, Zabrze, 41-819, Poland

*Corresponding author: Alicja Utrata-Wesołek; e-mail: autrata@cmpw-pan.pl; tel.: +48-32-271-077 (ext. 261)

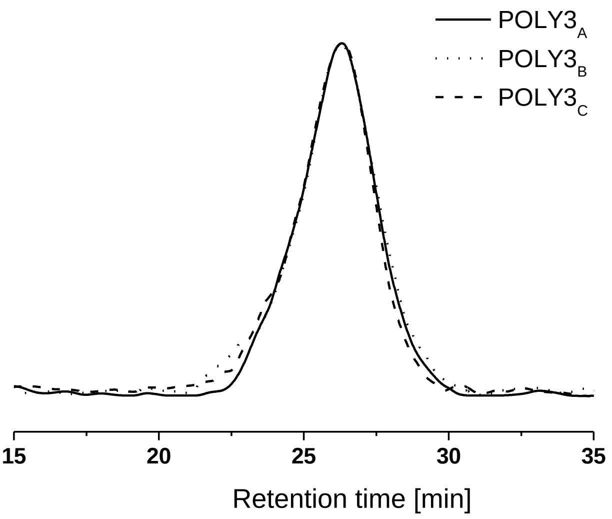

Figure S1. GPC-MALLS chromatograms for a representative poly[oligo(ethylene glycol) methacrylate] (POLY 3_A), its derivative containing OLA (POLY 3_B) and derivative containing OLA ended with acrylate groups (POLY 3_C)).

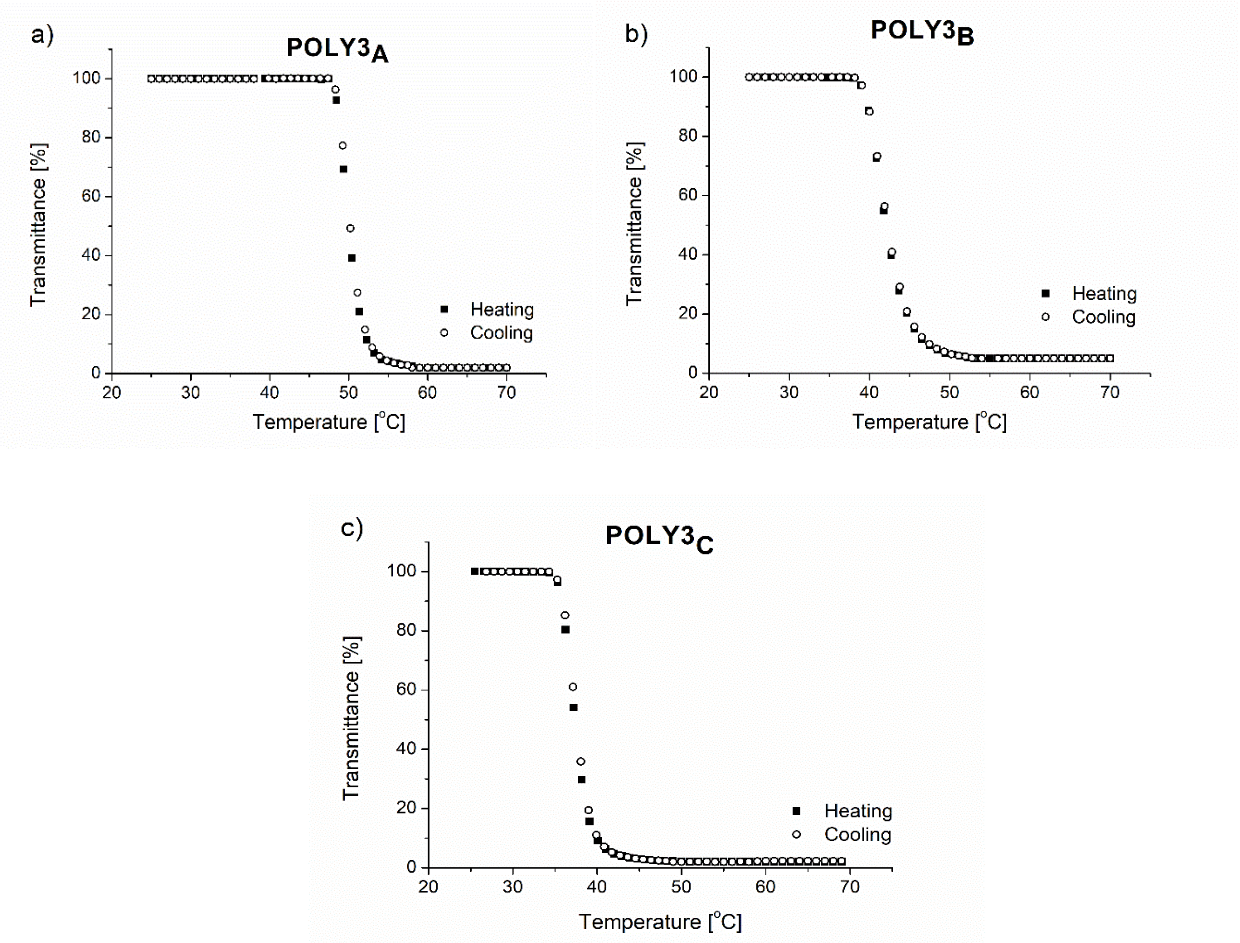

Figure S2. The heating–cooling cycles for (a) POLY 3_A, (b) POLY 3_B, (c) POLY 3_C solutions in water (1 mg/mL).

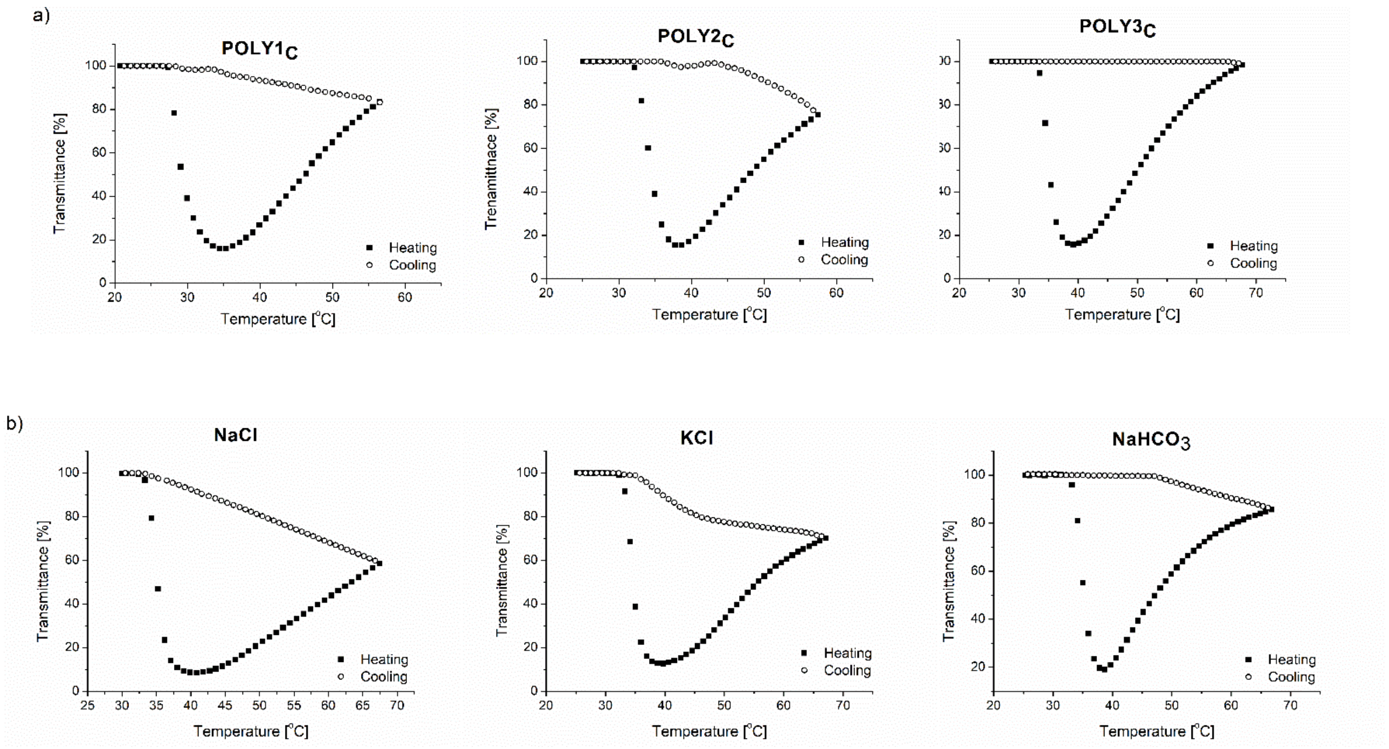

Figure S3. The heating-cooling cycles for (a) various POEGMA copolymers modified with OLA and acrylates in DMEM; (b) POLY 3_C copolymer in different salts.
